# The Discordance of Gene Mutations between Circulating Tumor Cells and Primary/Metastatic Tumor

**DOI:** 10.1016/j.omto.2019.08.006

**Published:** 2019-08-29

**Authors:** Qi Wang, Lanbo Zhao, Lu Han, Xiaoqian Tuo, Sijia Ma, Yiran Wang, Xue Feng, Dongxin Liang, Chao Sun, Qing Wang, Qing Song, Qiling Li

**Affiliations:** 1 Department of Obstetrics and Gynecology, First Affiliated Hospital of Xi’an Jiaotong University, Xi’an, Shaanxi 710061, China; 2 Center for Single-Cell Biology, First Affiliated Hospital of Xi’an Jiaotong University, Xi’an, Shaanxi 710061, China; 3 Cardiovascular Research Institute, Morehouse School of Medicine, Atlanta, GA 30310, USA

**Keywords:** CTC, nutations, heterogeneity, clinical utility

## Abstract

Circulating tumor cells (CTCs) are an important part in the field of “liquid biopsy.” However, major questions remain to be answered whether the mutations in the CTCs represent the mutations in primary tumor tissue and metastatic tumors. We compared the genetic mutations between CTCs and their matched tumors, and extracted data on the heterogeneity of the mutational status in CTCs and the change in mutations of CTCs before and during treatment. For mutations detected in single genes, we calculated the concordance of the mutations between the CTCs and primary tumor tissue. For mutations detected in multiple genes, we calculated the concordance of the mutations between the CTCs and primary/metastatic tumor tissue. The heterogeneity of the mutational status is clearly present in CTCs. For mutations detected in a single gene, the overall concordance of mutations is 53.05%. For mutations detected in multiple genes, the concordance of mutations is extremely different. The heterogeneity of the mutational status existed in single CTCs, and the mutational status of CTCs was discordant with that of tumor tissue.

## Introduction

Cancer has been one of the leading causes of death worldwide.^1^ With precision medicine becoming more popular for treating cancers, non-invasive methods have gradually aroused everyone’s attention and interests^2^ for the diagnosis and treatment of tumors. It is unrealistic for traditional tissue biopsy to capture spatial and temporal heterogeneity during tumor evaluation, and this fact is being considered as one of the major reasons for the failure of systemic cancer treatments. First, it is difficult or impossible to obtain tumor tissues at multiple time points. Second, some tumor tissues in specific locations might be not accessible for biopsy, and the surgical procedure might increase the risk of cancer “seeding” to other sites.^3^, ^4^, ^5^ Third, extreme heterogeneity is discovered in primary and metastatic tumors, which indicates the bias of a single biopsy.^6^ Therefore, tissue biopsy is unable to meet the needs for diagnosis and treatment. “Liquid biopsy,” which is a new approach that has emerged in recent years, may be able to solve these issues.

With the rapid advances of technologies, liquid biopsy has started to enter the clinic. Circulating tumor cells (CTCs) are the main features in the field of “liquid biopsy.” CTCs are approved by the US Food and Drug Administration (FDA) to aid in monitoring the prognosis of patients. A number of CTCs have been applied for detecting metastatic breast cancer,^7^ prostate cancer,^8^ colon cancer,^9^ ovarian cancer,^10^ esophageal cancer,^11^ bladder cancer,^12^ and other cancers. Beyond cell counting, the genotypic characterization of CTCs has gained increasing attention. For example, a series of mutations on the *TP53* gene was detected in CTCs from colorectal carcinoma patients in 2000.^13^ *TP53* mutations were detected in CTCs from metastatic triple-negative breast cancer patients.^14^ Mutations on the epidermal growth factor receptor (*EGFR*) gene were detected in CTCs of non-small-cell lung cancer.^15^ In the meantime, with the rapid development of next-generation sequencing (NGS), there is a chance for using CTCs in high-throughput molecular diagnosis. Moreover, because intratumor heterogeneity has been found in many tumor tissues, including renal cell carcinoma, breast cancer, lung cancer, prostate cancer, leukemia, esophageal squamous cell carcinoma, and others,^16^, ^17^, ^18^, ^19^, ^20^ a subsequent question is whether CTCs could reveal the genetic information of tumors. To address this question, we analyzed the sequence profiles of CTCs and their corresponding primary/metastatic tumors, and refined some gene mutation heterogeneities in this study.

## Results

### The Comparison between CTCs and Matched Primary Tumors in Single Genes

In lung cancer, the concordance of *EGFR* mutation is 75.27% (79/93) and the concordance of *KRAS* mutations 73.77% (135/183). In colorectal cancer, the concordance of *KRAS* mutation is 56.10% (92/164), and the concordance of *BRAF* mutations 46.15% (6/13). In melanoma, the concordance of *BRAF* mutations is 80.00% (52/65). In breast cancer, the concordance of *PIK3CA* mutation is 13.73% (28/204). We collected data from the above studies and calculated the total concordance of single mutations between CTCs and matched primary tumor as 53.05% (383/722) (Table 1; Table S1).

**Table 1:** Comparison of the Mutations in CTCs with Correspondent Primary Tumor Tissue in Single Genes in Different Subgroups

	No. of Patients	CTC^+^PTT^+^	CTC^+^PTT^−^	CTC^−^PTT^+^	Concordance
Total	722	383	117	222	0.530

Disease					

Melanoma	65	52	8	5	0.800
Lung cancer	276	205	27	44	0.743
Colorectal cancer	177	98	30	49	0.554
Breast cancer	204	28	52	124	0.137

Mutation					

*EGFR*	93	70	5	18	0.753
*BRAF*	78	58	8	12	0.744
*KRAS*	347	227	52	68	0.654
*PIK3CA*	204	28	52	124	0.137

Country					

China	192	155	15	22	0.807
The Netherlands	15	2	4	9	0.133
Greece	195	48	31	116	0.246
United States	55	33	9	13	0.60
Singapore	37	13	7	17	0.351
France	75	56	12	7	0.747
Italy	39	32	1	6	0.821
Germany	62	21	25	16	0.339
UK	28	10	9	9	0.357
Japan	7	6	1	0	0.857
Brazil	9	5	2	2	0.556
Spain	8	2	1	5	0.250

Method					

Magnetically sensed antibody sandwich assays	4	4	0	0	1.000
Scorpion amplification refractory mutation system technology	20	19	0	1	0.950
Whole-genome amplification and Sanger sequencing	10	9	1	0	0.900
RELP-PCR	34	29	3	2	0.853
NGS	37	31	0	6	0.838
Membrane arrays	190	153	15	22	0.805
Immunohistochemistry	59	46	8	5	0.780
dd-PCR	16	10	4	2	0.625
The peptide nucleic acid (PNA)-mediated PCR clamp with TaqMan-MGB allelic discrimination assays	13	5	2	6	0.385
PCR sequencing	157	51	52	54	0.325
Quantitative real-time PCR	14	3	5	6	0.214
Melting analysis	168	23	27	118	0.137

The concordance rate of mutations between CTCs and matched primary tumor was as follows: sum of patients with mutation existing in both CTCs and tumor tissue/sum of all patients. CTC, circulating tumor cell; dd-PCR, digital PCR; NGS, next-generation sequencing; PTT, primary tumor tissue; RELP-PCR, restriction fragment length polymorphism-PCR.

We next performed subgroup analyses of the data and concluded that melanoma (80%) and lung cancer (78.28%) had a relatively high concordance in CTCs and matched primary tumors. The mutations in *EGFR*, *KRAS*, and *BRAF* had high concordance values of 75.27%, 65.42%, and 74.36%, respectively. When analyzing a study from a different country, we reached a rough conclusion that the patient from a different country had extremely different concordance of mutations in CTCs and matched primary tumors. When comparing the different analysis methods, we observed that the concordances were all more than 90% by using magnetically sensed antibody sandwich assays, scorpion amplification refractory mutation system technology, whole-genome amplification (WGA), and Sanger sequencing (Table 1).

### The Heterogeneity of the Mutational Status in Single Genes

In colorectal cancer, the *KRAS (p.G13D)* mutation and *KRAS (p.G12D)* mutation were observed from two CTCs samples isolated from the same patient.^21^ In a study, six of nine *PIK3CA*-mutated CTCs from one patient carried the *PIK3CA (p. E545A)* mutation, whereas the *PIK3CA (p. E542K*) mutation was present in three of nine CTCs.^22^ In breast cancer patients, heterogeneity of the *PIK3CA* mutational status was widely discovered. A study revealed one patient with three different *PIK3CA* mutations in single CTCs but wild-type *PIK3CA* status in pooled CTC samples.^23^ Different ESR1 mutations in a single CTC were also tested in breast cancer.^24^ In a similar study, six single CTCs were isolated from a metastatic triple-negative breast cancer patient, where one CTC exhibited the same *TP53* (*R110 delG*) mutation and one revealed a *TP53 (R110 delC)* mutation; the remaining four single CTCs possessed the wild-type *TP53* sequence.^14^ Moreover, mutational heterogeneity of the *PIK3CA*, *TP53*, *ESR1*, and *KRAS* genes was revealed in forty individual epithelial cell adhesion molecule (EpCAM)-positive CTCs from five patients with metastatic breast cancer.^25^ In addition, with some CTCs from inflammatory breast cancer patients harboring different combinations of mutated and wild-type genes, intra-patient CTC mutation heterogeneity was confirmed.^26^ In lung cancer, double or multiple *EGFR* mutations were observed in CTCs of patients with non-small-cell lung cancer, which indicated CTC mutation heterogeneity.^27^, ^28^ In pancreatic cancer, the presence of various *KRAS* mutations in codons 12 and 13 in CTCs indicated heterogeneity.^29^

### The Heterogeneity of the Mutational Status in Multiple Genes

**Figure 1:**
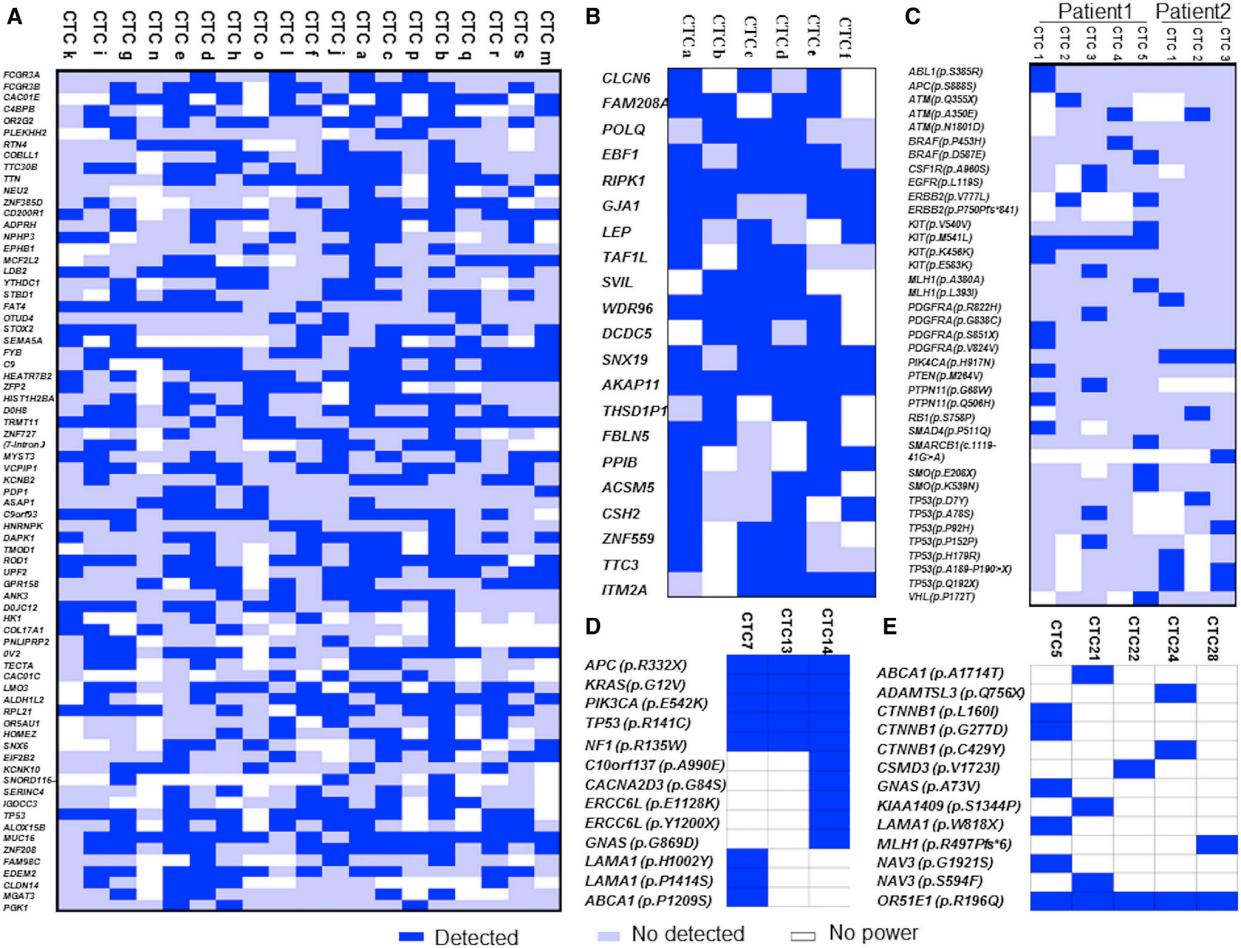
The Heatmap of the Mutations Detected in CTCs (A) Detection of mutations in 19 CTCs of metastatic prostate cancer patient 36 by whole-exome sequencing in Lohr et al.’s^32^ research. (B) Detection of mutations in six CTCs of metastatic prostate cancer patient 10 by whole-exome sequencing in Lohr et al.’s^32^ research. (C) Detection of mutations in five CTCs of lung cancer patient 1 and mutations in three CTCs of lung cancer patient 2 by single-sell exome sequencing in De Luca et al.’s^30^ research. (D) Detection of mutations by massive parallel sequencing in three CTCs of colorectal cancer patient 6 in Heitzer et al.’s^31^ research. (E) Detection of mutations by massive parallel sequencing in five CTCs of colorectal cancer patient 26 in Heitzer et al.’s^31^ research. The areas shaded in dark blue represent the mutations were obtained; the blank areas represent underpowered. The mutated genes are listed in the left column.

By collecting data from three experimental results and making a heatmap, we discovered that the mutational status in different CTCs had major differences (Figure 1).^30^, ^31^, ^32^ From the heatmap made from Lohr et al.’s^32^ research, we concluded that only two single-nucleotide variant (SNVs), *RIPK1* and *AKAP11*, were ubiquitous among all six sequenced CTCs collected from patient 10, and that no SNV shared in all 19 sequenced CTCs (Figures 1A and 1B). Except for the *KIT(p.M541L)* mutation from the CTCs of patient 1 and the *PDGFRA(p.V824V)* mutation from the CTCs of patient 2, the other mutations of CTCs in metastatic breast cancer represented extreme uniformity (Figure 1C).^30^ Another study certified that the *APC (p.R332X)* mutation, *KRAS (p.G12V)* mutation, *PIK3CA (p.E542K)* mutation, *TP53 (p.R141C)* mutation, and *NF1 (p.R135W)* mutation were ubiquitous in all CTCs of patient 6, and the *OR51E1 (p.R196Q)* mutation was shared in all CTCs of patient 26; the other mutations demonstrated great heterogeneity (Figures 1D and 1E).^31^

### The Concordance of Mutations between CTCs and Primary Tissues in Multiple Genes

**Figure 2:**
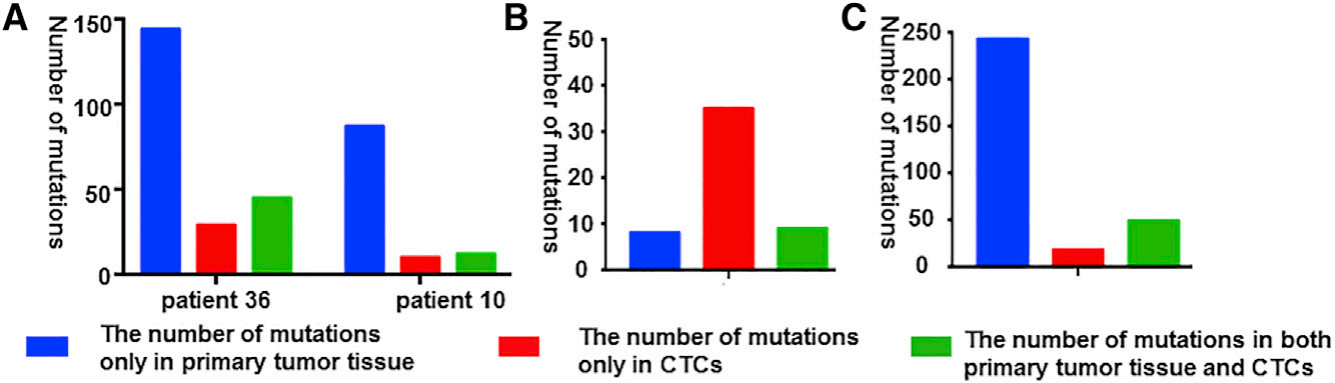
Comparison of the Mutations in Single/Pooled CTCs with Correspondent Primary Tumor Tissue by Whole-Exome Sequencing (A) The results in patients 36 and 10 of comparing the mutations in CTCs with correspondent primary tumor tissue by whole-exome sequencing in Lohr et al.’s^32^ research. (B) The results of comparing the mutations in CTCs with correspondent primary tumor tissue by whole-exome sequencing in Ni et al.’s^33^ research. (C) The results of comparing the mutations in CTCs with correspondent primary tumor tissue by whole-exome sequencing in Kong et al.’s^35^ research.

**Figure 3:**
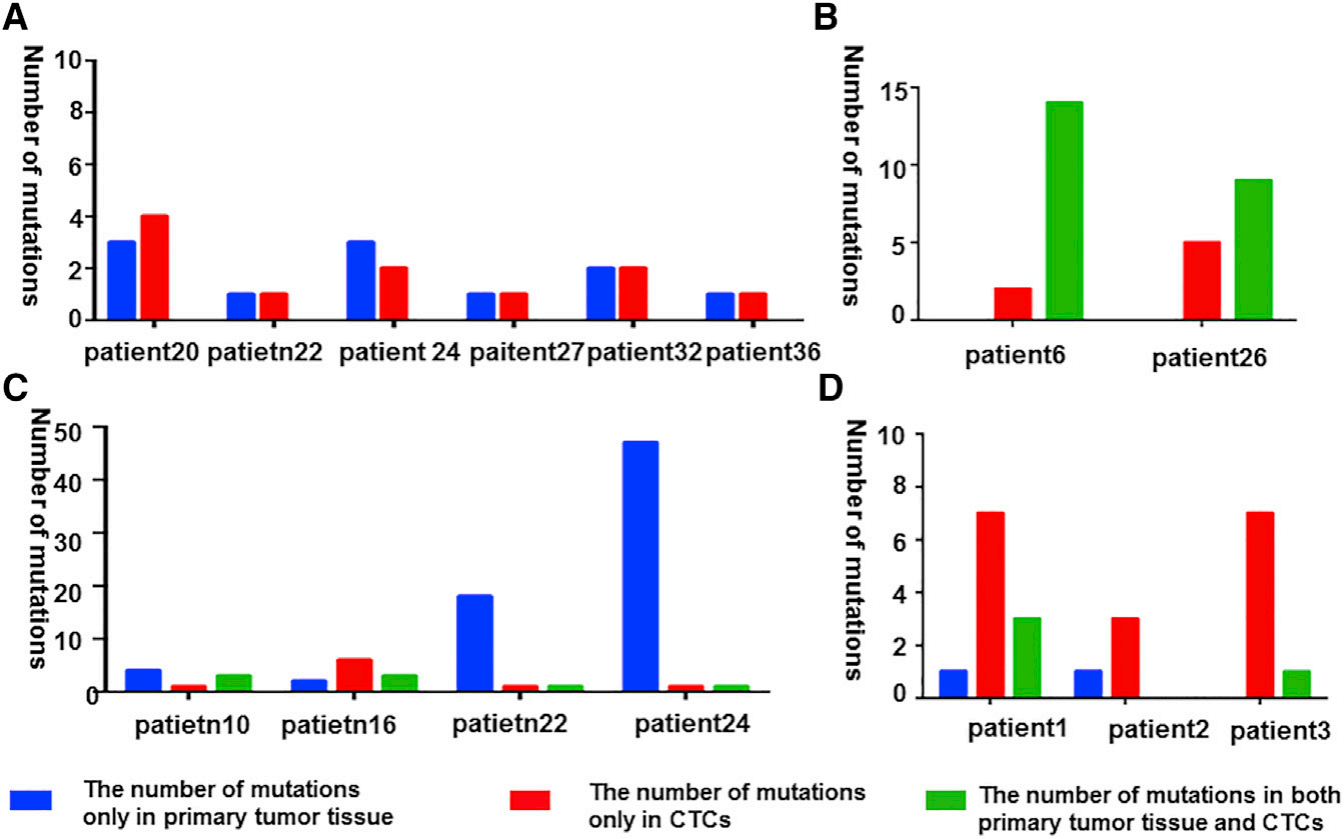
Comparison of the Mutations in CTCs with Correspondent Primary Tumor Tissue by Target Sequencing (A) The results comparing the mutations in CTCs with corresponding primary tumor tissue by target sequencing in Yoo et al.’s^34^ research. (B) The results comparing the mutations in CTCs with corresponding primary tumor tissue by target sequencing in De Luca et al.’s^30^ research. (C) The results comparing the mutations in CTCs with corresponding primary tumor tissue by target sequencing in Heitzer et al.’s^31^ research. (D) The results comparing the mutations in CTCs with corresponding primary tumor tissue by target sequencing in Lack et al.’s^36^ research.

We analyzed data from studies of single CTCs by NGS and compared the mutations in CTCs and the primary tissues, and validated extreme discordance. After analyzing the research of Lohr et al.,^32^ who sequenced 9 primary tissues plus 19 CTCs in patient 36, and 11 primary tissues plus nine CTCs in patient 10, we observed that 144 mutations were unique to primary tissues and 29 mutations were shared only among CTCs. Only 45 mutations were shared in both CTCs and primary tissues in patient 36. In another patient, only 12 mutations were shared in both CTCs and primary tissues, with 87 mutations in tumor tissues and 10 mutations in CTCs alone (Figure 2A; Table S2).^32^ The other researchers sequenced one primary tissue and nine CTCs using whole-exome sequencing. They revealed relatively more unique mutations, with only 35 mutations in CTCs, 8 mutations in primary tissues alone, and 9 mutations in both of them (Figure 2B; Table S2).^33^ In research using target sequencing, one study concerned with mutations in the Catalogue of Somatic Mutations in Cancer (COSMIC) validated that there were no mutations that matched in both CTCs and primary tissues from six patients, which indicated great discordance (Figure 3A; Table S3).^34^ Four mutations were shared in both CTCs and primary tissues, with 16 mutations present in only primary tissues and 10 mutations present in only CTCs after combining all mutations from three patients (Figure 3B; Table S3).^30^ In another study, the mutations were extremely different in four patients. The majority of the mutations were found only in the primary tumor tissues in patients 22 and 24 (Figure 3C; Table S3).^35^ However, one study observed a high consistency of mutations between CTCs and primary tissues. When they performed ultra-deep sequencing of the primary tumors, the researchers found that some unique mutations only in CTCs were shared in primary tissues, with the SNVs qualified with the deep SNV algorithm (Figure 3D; Table S3).^31^ Except for the single-CTC sequencing, pooled CTC sequencing was recently performed by researchers. Lack et al.^36^ isolated EpCAM^−^/CD45^+^ cells, divided them into three equal pools of 500 cells, and then performed WGA through multiple displacement amplification (MDA), with sequencing performed on a HiSeq2000. However, authors compared mutations in pooled CTCs with mutations in matched treatment-naive tumor tissue and/or castration-resistant tumor tissue without clearing out the synonymous mutations. In this study, we removed the synonymous mutations and merely analyzed the non-synonymous and stop-gain mutations. After reprocessing the data, we concluded that 18 mutations were uniquely possessed in CTCs and 249 mutations were discovered in only matched treatment-naive tumor tissues and/or castrate-resistant tumor tissues, with 29 mutations sharing in both of them (Figure 2C; Table S2).^36^

### The Concordance of Mutations between CTCs and Metastatic Tissues

**Figure 4:**
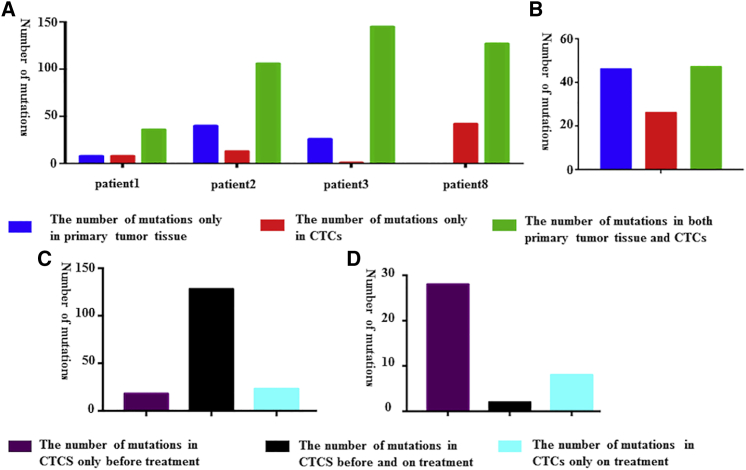
Comparison of the Mutations in CTCs with Correspondent Metastatic Tumor Tissue and Comparison of the Mutations in CTCs before and during Treatment (A) The results comparing the mutations in CTCs with corresponding metastatic tumor tissue in Ni et al.’s^33^ research. (B) The results comparing the mutations in CTCs with corresponding metastatic tumor tissue in Lohr et al.’s^32^ research. (C) The results comparing the mutations in CTCs before and during treatment in De Luca et al.’s^30^ research. (D) The results comparing the mutations in CTCs before and during treatment in Yoo et al.’s^34^ research.

In metastatic prostate cancer, Lohr et al.^32^ performed whole-exome sequencing in 19 individual CTCs and compared the mutations with matching metastatic lymph nodes. We analyzed the data and came to the conclusion that 26 mutations were present in CTCs alone and 46 mutations were unique to metastatic tissues, with only 45 mutations in both CTCs and metastatic tumors tissues (Figure 4B; Table S4).^32^ In a similar lung cancer study, researchers sequenced mutations in individual CTC and metastatic tumors tissue in four patients, and observed that the majority of the mutations were shared between CTCs and metastatic tumor tissue (Figure 4A; Table S4).^33^

### The Genetic Shift of CTCs during Treatment

Given that repeat biopsy is undesirable, the ability to detect mutations in CTCs over time is important for monitoring the evolution of disease during the treatment. Independent of disease types, treatment methods, treatment time, and the detecting method, we analyzed data from two studies and reached opposite results. Two of the 38 mutations were always present in CTCs in one study, but the other study indicated that 128 out of 169 mutations were present all the time (Figures 4C and 4D; Table S5).^30^, ^34^

## Discussion

### The Different Definitions Caused by Amplification and Sequencing Brought Errors in Analyzing the Mutations Sequenced by NGS

For single-cell mutation analysis, it has been suggested that errors can be generated during whole-genome amplification and sequencing, which lead to high false discovery ratio (2–3 × 10^−5^) and high allele dropout ratio (11%).^31^ To eliminate the mutations introduced by amplification and sequencing errors, Ni et al.^33^ sequenced eight single CTCs from one patient, and only when an SNV in a CTC was detected in two other CTCs or in primary/metastatic tumors was it called a mutation. In another study, Lohr et al.^32^ sequenced 19 single CTCs from one patient and 6 single CTCs from another patient; they think that if a SNV in a CTC was detected in two other CTCs libraries, it will be called a mutation. On the other hand, Heitzer et al.^31^ do not agree with the view that a SNV should be defined as a true mutation only if it occurs in a certain number of cancer cells, because the available CTCs are often rare but of high quality. De Luca et al.^30^ trust high-depth and high-coverage sequencing; they sequenced CTCs with a mean depth of 1,500×, ranging from 1,046× to 2,478× depth of coverage for each amplicon per sample by Ion Torrent. Defining mutations after sequencing can lead to substantial controversies, and the methodological consensus must be unified.

To eliminate the artifacts caused by amplification and sequencing, it may be necessary for single-cell (not only in CTCs) analyses to sequence multiple single cells,^37^ such as at least three cells, to confirm the mutations in tumors.^38^ With the mutations correctly called, bioinformatics could play more important roles in the diagnosis and prognosis of cancer.^39^

### The Possible Reasons for the Heterogeneity of Mutational Status

The limitations of the amplification and sequencing method might cause the heterogeneity. Besides, using different sequencing methods might yield different mutational results. Comparing the performance of high-resolution melt (HRM), allele-specific PCR (ASPCR), and pyrosequencing, Mohamed Suhaimi et al.^40^ observed that the mutation status sequenced by above methods were different.

In addition to the limitations of the amplification and sequencing methods, two other reasons may also explain the heterogeneity of the mutational statuses of single CTCs. There is an opinion that CTCs disseminated into the blood from primary tumors and/or metastatic tissues represent a surrogate for the tissue-based tumor, and analyses of CTCs might provide insight into tumor tissue heterogeneity. Therefore, the heterogeneity of the mutational status of single CTCs might be caused by the heterogeneity of the mutational status of single primary/metastatic tumor cells. Besides, some researchers first performed genome sequencing in which only the *PIK3CA* mutation was possessed in the cultured CTCs. When those cultured CTCs were sequenced, some newly acquired mutations in the *ESR1* gene, *PIK3CA* gene, and *FGFR2b* gene were observed.^41^ Therefore, we suggest that the change in CTCs and their microenvironment, including epithelial-to-mesenchymal transitions,^42^ mesenchymal-to-epithelial transitions,^43^ blood flow, and immune cell attack might alter the mutations.

Finally, research about acquired mutation between targeted treatments reveals another potential reason for the heterogeneity of mutational status. For example, Blakely et al.^44^ confirmed increasing heterogeneity of an *EGFR* mutant cancer across multiple lines of therapy. Similarly, Piotrowska et al.^45^ showed the heterogeneity and coexistence of *T790M* and *T790* wild-type resistant subclones drive mixed response to third-generation *EGFR* inhibitors in lung cancer. Thus, the subclonal acquisition of mutation during therapy might cause the heterogeneity of mutational status and discordance of mutations between CTCs with primary and/or metastatic tumor tissue.^46^

### The Probable Reasons for the Discordance of Mutations between CTCs with Primary and/or Metastatic Tumor Tissue

It is necessary to elucidate the reasons for the discordance observed between primary/metastatic tumor tissues and CTCs. Several potential reasons have been proposed. First, limitations of current isolating and sequencing techniques may hamper the identification of the entire spectrum of mutational statuses and causes of the discrepancies.^41^ Second, with subcolonies and heterogeneity within the entire tumor tissue considered, certain numbers of CTCs are unlikely to contain the entire spectrum of mutations. When the CTCs isolating and enriching method improves to obtain sufficient numbers of CTCs, people might confirm if the discrepancies in the mutation statuses between tumor/metastatic tissues and CTCs are caused by the subcolonies and heterogeneity in bulk tumor tissue.^16^, ^47^ Third, the CTCs isolation technologies, including Cell Search, which is based on EpCAM, may have substantial limitations.^48^, ^49^ Many studies conclude that primary tumor cells should undergo epithelial-to-mesenchymal transition (EMT) to invade the blood vessels, which accelerates the production of CTCs. However, some technology depends on the EpCAM marker for the isolation of CTCs, which might ignore the EMT-transformed CTC.^48^, ^49^ To solve these problems, it has been suggested that using other antibodies or different enrichment systems to deplete normal blood cells and enrich the CTCs may be a solution.^49^, ^50^ Finally, the opinion that CTCs can acquire private and unique mutations during the evolution of the tumor has been proposed by some researchers, which might cause the large heterogeneity in CTCs and the discrepancies in mutation statuses between the tumor or metastatic tissues and CTCs.^41^, ^51^ A high proportion of concordance has been reported by most studies with mutations between CTCs and tumor tissues or metastatic tissues, which suggests the potential usefulness of using CTCs to profile mutations of tumors.

Minimally invasive access to CTCs offers a unique opportunity to monitor disease progression and guide drug management.^52^ However, amplification and sequencing errors, the lack of a uniform definition of mutations in the sequenced single CTCs, the heterogeneity of the mutational status in single CTCs, and the discordance of mutations between CTCs with primary and/or metastatic tumor tissue must be explored before CTCs can be widely used in clinics.

## Materials and Methods

### The Concordance Compared between CTCs and Primary Tissues in Single Genes

We retrieved in PubMed the studies comparing certain single-gene mutations between CTCs and primary tissues in many patients. The following data were extracted: the number of patients with the mutation shared in the CTCs and matched primary tumor, the number of patients with the mutation discovered in CTCs, but not in the matched tumors, and the number of patients with the mutation carried in the matched tumors, but not in CTCs. Then, we calculated the concordance rate of mutations between the CTCs and matched tumors as follows: sum of patients with mutation matched in both CTCs and primary tissues/sum of all patients. The data in different subgroups were also compared for the concordance of mutations.

### The Concordance Compared between CTCs and Primary Tissues in Multiple Genes

We searched PubMed and collected the articles pertaining to multiple mutations (a larger number of mutations variants in at least 10 genes) sequenced by NGS. Then, we collected data about the comparison of the mutations between CTCs and primary tissues using NGS. Because of the different definitions of mutations in different studies, we gathered the mutations that were exclusively in CTCs, exclusively in primary tissues, and in both CTCs and primary tissues. The concordance rate of mutations between CTCs and matched primary tissues was calculated as follows: sum of mutations in both CTCs and primary tissues/sum of all mutations.

### The Heterogeneity of Mutational Status in Multiple Genes

We searched PubMed and collected data comparing the mutations in different CTCs of the same patient using NGS. Then, we extracted the data about heterogeneity of mutational status and created a heatmap.

### The Discordance between CTCs and Metastatic Tissues on Genetic Mutations

Studies comparing the mutations between CTCs and metastatic tissues using NGS were searched and collected in PubMed. Because of the different definitions of mutations in different studies, we gathered the mutations present exclusively in CTCs, exclusively in metastatic tissues, and in both CTCs and metastatic tissues. Then the concordance rate of mutations between CTCs and matched metastatic tissues was calculated as follows: sum of mutations in both CTCs and metastatic tissues/sum of all mutations.

### The Genetic Shift of CTCs during Treatment

We searched PubMed and collected the studies comparing the mutations in CTCs before and during treatment using NGS. Because of the different definitions of mutations in different studies, we gathered information about the mutations that were confirmed exclusively in CTCs before treatment, exclusively in CTCs during treatment, and in CTCs both before and during treatment.

## Author Contributions

Q.S. and Q.L. designed and configured this study. Qi Wang and L.Z. performed the majority of the experiments. L.H., X.T., S.M., and Y.W. prepared the figures. Qi Wang wrote the manuscript. X.F., D.L., C.S., and Qing Wang helped to revise the manuscript.

## Conflicts of Interest

The authors declare no competing interests.

## Supplemental Information

Table S1. Comparison of the Mutations in CTCs with Corresponding Primary Tumor Tissue in Single Genes

Table S2. Comparison of the Mutations in Single/Pooled CTCs with Corresponding Primary Tumor Tissue by Whole-Exome Sequencing

Table S3. Comparison of the Mutations in Single CTC with Correspondent Primary Tumor Tissue by Target Sequencing

Table S4. Comparison of the Mutations in Single CTC with Correspondent Metastatic Tumor Tissue

Table S5. Comparison of the Mutations in CTCs before and during Treatment
